# Using Hemoglobin A1C as a Predicting Model for Time Interval from Pre-Diabetes Progressing to Diabetes

**DOI:** 10.1371/journal.pone.0104263

**Published:** 2014-08-05

**Authors:** Chen-Ling Huang, Usman Iqbal, Phung-Anh Nguyen, Zih-Fang Chen, Daniel L. Clinciu, Yi-Hsin Elsa Hsu, Chung-Huei Hsu, Wen-Shan Jian

**Affiliations:** 1 Division of Endocrinology and Metabolism, Department of Internal Medicine, Taipei Medical University Hospital, Taipei, Taiwan; 2 Graduate Institute of Biomedical Informatics, College of Medical Science and Technology, Taipei Medical University, Taipei, Taiwan; 3 School of Health Care Administration, Taipei Medical University, Taipei, Taiwan; 4 Translational Medicine Program, College of Medical Science and Technology, Taipei Medical University, Taipei, Taiwan; 5 Department of Nuclear Medicine, Taipei Medical University Hospital, Taipei, Taiwan; Boston University, United States of America

## Abstract

**Objective:**

The early identification of subjects at high risk for diabetes is essential, thus, random rather than fasting plasma glucose is more useful. We aim to evaluate the time interval between pre-diabetes to diabetes with anti-diabetic drugs by using HbA1C as a diagnostic tool, and predicting it using a mathematic model.

**Methods:**

We used the Taipei Medical University Affiliated Hospital Patient Profile Database (AHPPD) from January-2007 to June-2011. The patients who progressed and were prescribed anti-diabetic drugs were selected from AHPPD. The mathematical model used to predict the time interval of HbA1C value ranged from 5.7% to 6.5% for diabetes progression.

**Results:**

We predicted an average overall time interval for all participants in between 5.7% to 6.5% during a total of 907 days (standard error, 103 days). For each group found among 5.7% to 6.5% we determined 1169.3 days for the low risk group (i.e. 3.2 years), 1080.5 days (i.e. 2.96 years) for the increased risk group and 729.4 days (i.e. 1.99 years) for the diabetes group. This indicates the patients will take an average of 2.49 years to reach 6.5%.

**Conclusion:**

This prediction model is very useful to help prioritize the diagnosis at an early stage for targeting individuals with risk of diabetes. Using patients' HbA1C before anti-diabetes drugs are used we predicted the time interval from pre-diabetes progression to diabetes is 2.49 years without any influence of age and gender. Additional studies are needed to support this model for a long term prediction.

## Introduction

Hemoglobin glycation A1C or glycosylated hemoglobin (HbA1C) was clinically first used 30 years ago to access the degree of chronic hyperglycemia among diabetic patients [1]. Diabetes is a highly morbid and highly cost disease due to its high incidence of macro and micro vascular complications [2]. In 2010, there were 26.8 million people with diabetes in the US, and it is estimated in 2030 the number of affected individuals would be 36 million [3]. In Taiwan, there were 2.1 million people with diabetes in 2010, 9.2% of the total population; the medical cost of diabetes accounts for 11.5% of the total medical expense, 4.3 times of non-diabetes [4]. Thus, it's important to consider screening for pre-diabetes in order to delay the diabetes onset and to reduce the micro-vascular and macro-vascular complications along with the socioeconomic burden.

The treatment guidelines for diabetes treatment as well as several studies regarding long-term complications revealed positive impact of the measurement of plasma glycosylated hemoglobin on the disease [5], [6]. The advantage of HbA1C measurements is not being affected by fasting or timed sampling and to show considerable within-day and day-to-day variations. Therefore, HbA1C seems more convenient and reproducible than blood glucose measurements [7], [8]. Most importantly, there are standardized, aligned to the Diabetes Control and Complications Trial (DCCT) methods of HbA1C measurements [9]. HbA1C measurements provide a practical alternative for screening diabetes and pre-diabetes states. Moreover, American Diabetes Association (ADA) and World health organization (WHO) experts has been recently proposed HbA1C as a diagnostic tool for detection of diabetes mellitus [10], [11]. ADA also recommended recently that HbA1C ≥6.5% is one of the diagnostic criteria of diabetes [12], and HbA1C from 5.7%–6.4% could help to identify individuals at high risk/increased risk for diabetes, and named the term as pre-diabetes [13], [14].

Although HbA1C is frequently seen in our daily practice, perplexities still exist in the interpretation of HbA1C results, and an optimal cut-off is still a point of critical debate [6], [15], [16]. The HbA1C time interval before diabetes diagnosed such as the interval between HbA1C 5.7% to 6.5% is still unclear [17]. Less attention has been given towards using HbA1C values to predict diabetes. By using prediction models it would help create characteristic models of physician decision strategies in the treatment of pre-diabetes populations of patients and to advance patient safety research [18].

To our knowledge, there have been no previous studies examining plasma A1C levels using a linear regression trend line model to estimate the average time interval from non-diabetes to diabetes with the start of anti-diabetic medicines. In this study, we sought to characterize patient's plasma A1C with regard to their low, increasing, high, and very high risk toward diabetes disease progression. Thus, the aim of this research was to study the progression of HbA1C before the diabetes is diagnosed, and to estimate the average time interval from non-diabetes to diabetes with anti-diabetic drugs. We evaluated the time interval between pre-diabetes and Diabetes (until taking medicine) by using hemoglobin A1C as a diagnostic tool.

## Research Design and Methods

### Study design

We identified outpatients who visited three hospitals - Taipei Medical University hospital (TMUH), Wan-Fang hospital (WFH), and Shuang-Ho hospital (SHH) from January 1, 2007 to June 30, 2011. We used the incident user design with follow-up for each patient beginning at the index date of first glycosylated hemoglobin (HbA1C) laboratory examination. All patients were followed up until their first anti-diabetic medications was prescribed at the end of June 2011. All laboratory examinations of patient (i.e. blood urea nitrogen (BUN), Cholesterol, Creatinine, Glucose AC, glutamic-oxalacetic transaminase (GOT), glutamic-pyruvic transaminase (GPT), high-density lipoprotein (HDL), low-density lipoprotein (LDL), Triglyceride, and Uric Acid) were considered if they were taken in during 3 months period before the index date. All treatment of patient (i.e. medication prescriptions) were also collected in the study.

### Patients and participants

**Figure 1:**
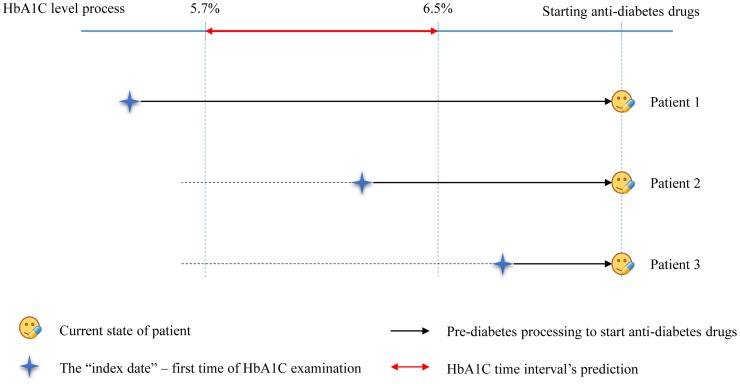
Comparative flowchart for HbA1C time intervals prediction in the study.

We identified 49,648 potentially eligible subjects which took glycosylated hemoglobin examination tests from claimed data (i.e. treatment history, laboratory test and medications prescribed) from three study hospitals. We excluded 39,762 patients that used anti-diabetic medications (see Appendix S1) before HbA1C tests. In addition, 8,607 patients were excluded due to once time tests found during study period in the hospital data. After applying the linear regression trend-line model to estimate the interval days of HbA1C level from 5.7% to 6.5% (the risk intervals for diabetes), we excluded another 27 patients found at 2.5% from both sides of the extreme intervals. Therefore, 1,252 patients were finally included in the study (see Appendix S2). Based on the index date value, all patients were categorized separately into three groups (low risk, increased risk and diabetes) for further statistical analysis [19] (see Figure 1).

#### Low risk

Patients with values less than 5.7% at index date.

#### Increased risk

Patients with values in between 5.7% to 6.5% at index date.

#### Diabetes

Patients with values higher than 6.5% at index date.

### Statistical Analysis

**Figure 2:**
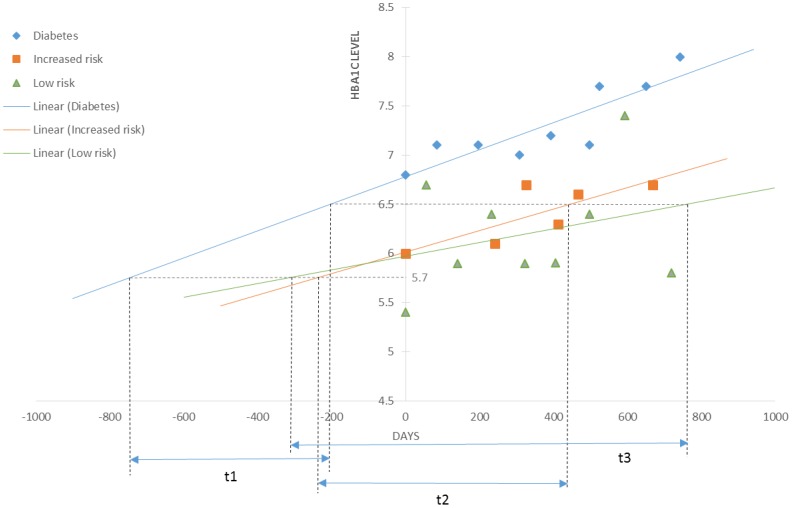
An example of linear regression trend-line prediction model used in the study.

One way ANOVA was used to compare each variable among all three group of patients. A p-value of less than 0.05 was considered to be significant for our analysis. A linear regression trend-line was calculated for every HbA1C value of each patient. The linear regression trend line was used to compute the intervals for diabetes risk and estimated the days by taking into account HbA1C from 5.7% to 6.5% for each patient included in the study. The mean for diabetes risk intervals was calculated within each group as well as overall for all three groups. An example is shown in figure 2 for interval detections by cutoff values with time periods. The t1, t2, t3 represent the risk intervals from pre-diabetes progressing to diabetes for diabetes, increased risk, and low risk patients, respectively. In the study, SPSS 20 integrated with R 2.14.0 was used to compute the linear regression trend line for each patient.

### Ethical approval

This study has been approved by the ethical committee of joint institutional review board, TMU-JIRB-201306005 from Taipei Medical University, Taiwan.

The institutional review board waived the need for written informed consent from the participants as patient records/information was anonymized and de-identified prior to our analysis.

## Results

Table 1 shows the characteristics of the total 1252 patients, with the mean age of 62.7 (SD = 13.0) years, out of which 53.26% were male. This table shows the characteristics of patients which were divided into three groups based on the index date of HbA1C progression for their clinical parameters measurement analysis. Out of all, the average of all these three groups 61.83% patients have tested GPT and 31.96% GOT, separately. All of them were under 41 mg/dl, which indicates that patients liver function were within normal limits/range and none was of clinical importance. Although 70.3% patients have their cholesterol checked all of them were found under 200 mg/dl, which also indicated that patients' total cholesterol levels were within normal limits. However, their high triglycerides, high LDL and low HDL results were high risk for the cardiovascular disease (CV), coronary heart disease, peripheral vascular disease, stroke, hypertension, dyslipidemia, and obesity.

**Table 1:** Display characteristics of patients by groups.

Characteristics	Low risk group (n = 74)	Increased risk group (n = 541)	Diabetes group (n = 637)	p-value
**Age, mean (SD)**	61.8 (13.8)	61.92 (12.8)	63.41 (13.0)	0.123
Age <45	35.6 (7.3)	37.9 (6.4)	38.2 (5.7)	
45≤Age<60	54.3 (4.1)	53.1 (3.7)	53.2 (4.0)	
60≤Age<75	68.1 (3.9)	66.2 (4.3)	67.2 (4.4)	
Age ≥75	81.9 (3.4)	80.3 (4.3)	80.6 (3.9)	
**Gender**				0.010
Male, n (%)	48 (64.9)	263 (48.6)	295 (46.3)	
Female, n (%)	26 (35.1)	278 (51.4)	342 (53.7)	
**BUN**				0.006
n (%)	20 (27)	115 (21.3)	145 (22.8)	
mean (SD) mg/dl	34.8 (27.3)	20.9 (16.0)	26.8 (22.1)	
**Cholesterol**				0.072
n (%)	47 (63.5)	406 (75.0)	461 (72.4)	
mean (SD) mg/dl	180.6 (46.1)	197 (55.6)	193.6 (39.2)	
**Creatinine**				0.006
n (%)	46 (62.2)	389 (71.9)	457 (71.7)	
mean (SD) mg/dl	1.8 (2)	1.1 (1.3)	1.5 (2)	
**Glucose AC**				<0.001
n (%)	63 (85.1)	458 (84.7)	529 (83)	
mean (SD) mg/dl	116 (22.4)	119 (20.2)	139.4 (42.6)	
**GOT**				0.901
n (%)	24 (32.4)	188 (34.8)	183 (28.7)	
mean (SD) mg/dl	34.9 (16.2)	33.2 (26.3)	32.7 (21)	
**GPT**				0.859
n (%)	40 (54.1)	373 (68.9)	398 (62.5)	
mean (SD) mg/dl	35.1 (31.1)	35.1 (31.1)	35 (33.5)	
**HDL**				0.064
n (%)	41 (55.4)	290 (53.6)	303 (45.6)	
mean (SD) mg/dl	38.7 (10.5)	43.4 (11.8)	42.9 (12.6)	
**LDL**				0.058
n (%)	12 (16.2)	141 (26.1)	182 (28.6)	
mean (SD) mg/dl	96.7 (26.6)	112.5 (29.9)	118 (37)	
**Triglyceride**				0.325
n (%)	51 (68.9)	421 (77.8)	478 (75)	
mean (SD) mg/dl	167.3 (145)	162.6 (126.4)	176.9 (157)	
**Uric Acid**				0.379
n (%)	31 (41.9)	271 (50.1)	303 (47.6)	
mean (SD) mg/dl	6.9 (1.9)	6.5 (1.4)	6.5 (1.7)	

In a nutshell, these patients were in potential risk of metabolic syndrome, and therefore the risk of primary cardiovascular outcome is increased. These lab test results showed that there are some parameters which might need close monitoring that could provide the rationale why physicians followed HbA1C of patients so often (Table 1).

In Table 2, we calculated the duration of medication (between the first HbA1C test/the index date and start taking medication) and interval detection days (the interval days a patient at least stayed in between 5.7% to 6.5% range). According to our linear regression trend line model the average interval of HbA1C progressing from 5.7% to 6.5% in all three groups found a total of 907 days. This shows that if a patient's HbA1C levels value is equal to or less than 5.7%, then it will take at least 2.49 years to increase HbA1C levels value up to 6.5%. However, HbA1C reveals how much sugar has been around for the preceding three months. In most cases, the normal range is 4%–5.9%. In poorly controlled diabetes, its 8.0% or above, and in well controlled patients it's less than 7.0%. The benefits of measuring HbA1C is that is gives a more detailed description of what is happening over the course of time (3 months), and the values do not bounce as much as finger stick blood sugar measurements. Using our model, we conclude that it could be possible to have this test at least after 2 years.

**Table 2:** Display the duration of medication and interval detection of study.

	Low risk group	Increased risk group	Diabetes group	Overall patients	p-value
**Duration medication (days)**					<0.001
**Mean**	641.6	640.9	565.7	602.7	
**SE**	37.8	14.0	12.2	9.0	
**Interval detection (days)**					0.211
**Mean**	1169.3	1080.5	729.4	907.1	
**SE**	331.9	176.4	130.6	103.1	

Note: SE, standard error.

When we divided patients into three categories, 1) diabetes (diabetic but not yet on anti-diabetic drugs), 2) increased risk (medium) and 3) low risk (slow) then slow and medium group would require the same amount of time as the fast progressing disease group which needs at least 729.4 days (i.e. 1.99 years) to reach the HbA1C levels up to 5.7% to 6.5%. Although, the speed of the disease progression was faster, it still took 2 years to reach the 6.5% levels. These results indicates that whether the disease progression is in slow, medium or at a faster progression stage, it would require almost a similar time to rise the HbA1C levels which we predict through our mathematical model. While we looked particularly at a low risk group (slow progressing diabetic) we found that it also requires 3.2 years to reach the 6.5% HbA1C levels (Table 2).

## Discussion

The linear regression trend line is commonly used in the economic fields [20]–[23], but it can also be successfully employed in medical informatics research for predicting uveal melanoma metastasis [24], in video-assisted thoracic surgery (VATS) [25] and some other studies [26], [27]. Our prediction model using statistical measures in considering the HbA1C values that manifest the classification ability of a model in listing an individual's approximate daily occurrences for becoming a diabetes patient and take diabetic drugs. We used the HbA1C reference values and divided patients into three groups on the basis of their disease progression. In this study, we defined the best cut-off point as the key indicator to identify the intervals within cut-off values to progression of diabetes. Our prediction model in this study shows that the cut-off points of HbA1C helpful to diagnose diabetes and could be applied generally on a population for diabetes identification progression purposes.

We found via our linear regression trend-line model that low risk group (slow progression) required 1169.3 days (i.e. 3.2 years) to reach the 6.5% HbA1C levels. Still, this level of glycated hemoglobin will change in the individuals if the physician should intervene at the right time. The 5.7% level of HbA1C is the cut-off point for the low risk diabetes progression in the investigated population. The best cut-off point for diabetes screening with the highest sum of sensitivity and specificity is an HbA1C level of 5.8% found in a Dutch population [28].

The median progression of diabetes also found increased risk patients with HbA1C values at an index in between 5.7% to 6.5% which required 1080.5 days (i.e. 2.96 years in increased risk group). This indicates that if the patients have levels in between 5.7% to 6.5%, they need at least 3 years to be considered diabetic and to get anti-diabetic prescriptions. However, this occurrence could be changed by changing their life styles. As we observed, many patients had 6.5% HbA1C but they did not start to take diabetic medicine which might be because of their life style. Some studies found that this can be attributed to the ability of A1C to predict the incidence of diabetes [6]. The incidence of diabetes progressively increased among patients with the level of HbA1C lower than recommended by ADA. Glycated hemoglobin value ≥5.0% was a substantial diabetes predictor and the risk significantly expanded when HbA1C was 6.0%–6.4% [29].

Our study shows that an HbA1C greater than 6.5%, which is also included by ADA as a one of the diagnostic criteria for diabetes. We found that the fast progressing diabetes group needs at least 729.4 days (i.e. 1.99 years) to cross the HbA1C levels 6.5% cut-off point. Although, the speed of the disease progression was faster, it still took 2 years to cross the 6.5% cut-off levels. In another study of a Dutch population, the HbA1C levels ≥6.0% were used to screen for diabetes in the general population [28]. Several studies were done on prediction of diabetes progression in different populations. In an Australian population the diabetes absence is predicted by using HbA1C ≤5.5% and HbA1C ≥7.0% levels which also predicts the presence of diabetes, while HbA1C 6.5%–6.9% levels is highly probable for diabetes [30]. In Asian Indians, studies found a value HbA1C cut-off point of 5.6% which optimally identified pre-diabetes, but was less than 70% accurate [31]. Considerable variations were observed between fasting plasma glucose and HbA1C- based diagnosis of diabetes and pre-diabetes in older population only. However, the differences are among racial, ethnic and gender groups [32], [33]. Some studies suggest that HbA1C may be a suitable diagnostic tool for diabetes if fasting plasma glucose or OGTT are not available [34].

For overall three groups, the average interval days detected for the levels to rise more than 6.5% requires almost 907.1 days (i.e. 2.49 years). Therefore, our results indicates that whether the disease progression is in slow, medium or at a faster stage, it would require almost similar times to rise the HbA1C levels which we predicted through our linear regression trend line in our model. Some studies show that HbA1C is a good diagnostic tool for diabetes incidence, however the optimal cut-off value is not generally accepted [35]. WHO experts found that there are currently insufficient evidences to make any formal recommendations on the interpretations of HbA1C levels and also underlined that diabetes diagnosis in asymptomatic individuals should not only rely on the basis of single HbA1C [36].

During our study we also found that even at an HbA1C greater than 6.5%, the patients did not have anti-diabetic drugs prescribed for up to 565.7 days (SE, 9 days). It indicates that HbA1C is not the only consideration for a physician when prescribing diabetic drugs but also includes other factors affecting patients such as hypertension, cardiovascular disease, body mass index (BMI) and life style. Although some studies demonstrated that HbA1C levels could predict the risk of micro vascular complications associated with diabetes, more evidence is needed to establish the HbA1C role as diagnostic tool for diabetes [6], [37], [38].

Selvin *et.al.* revealed in 2011 that HbA1C is a good predictor for diabetes and could be used as a gold standard for diabetes diagnosis [35]. The advantage of the HbA1C is it does not require fasting to check for glucose level like Ac glucose. For that, patients should be in fasting with at least 8 hours but for hemoglobin measurement, it could be taken any time. Thus, it is more convenient to check HbA1C then Glucose Ac. Although, our model can predict the interval of days accurately, however, the time interval could be changed according to the life style changes. Williamson et.al in 2010 concluded that about 30% of the U.S. adult population had pre-diabetes symptoms, but only 7.3% were aware they had it [39]. Therefore, we conclude that health care providers would be able to intervene at patients' early progression stages on the basis of these interval days and patients could delay diabetes progression just by changing their life style behavior [40]. If patients start exercising and carefully monitor their diet, it could delay their diabetes progression and could delay the micro and macro vascular complications.

We selected patients with end point of taking diabetes drugs by using a five years TMU Affiliated Hospital Patient Profile Database (AHPPD). The short duration of study could affect the study results generalization. Another limitation we might think about is anemic patients which we did not considered in our study. Furthermore, we did not have complete blood cell count (CBC) examination results, Blood pressure, Body Mass Index (BMI) and family history in our data base which would also be considered as limitation as it might influence some factors but not to a large extent.

This prediction model could identify DM status at early stage that would be helpful for health care practitioners to alert their patients and start an intervention regarding changing their life style which could delay or prevent onset of diabetes. The average time interval detected to cross the 6.5% levels would require at least 2.49 years, enabling an early detection to make patients aware. Efforts should be made to improve awareness of pre-diabetes such as exercise and diet awareness, to increase promotion of healthy behaviors, and to improve availability of evidence-based lifestyle programs needed to slow the progression of new cases of diabetes. On the other hand, it is also important for policy makers to consider this important issue and suggest an HbA1C fee exempt exam within the critical two- year time frame in order to better aid and manage this long term heavy burden disease.

## Supporting Information

Appendix S1 **Detail of anti-diabetes used in three hospitals in this study.**
(DOCX)

Appendix S2 **Overall study design.**
(DOCX)
