# Supplementary material for: Using Hemoglobin A1C as a Predicting Model for Time Interval from Pre-Diabetes Progressing to Diabetes
Source: PLoS One. 2014 Aug 5;9(8):e104263. doi: 10.1371/journal.pone.0104263 (PMC4122428; doi:10.1371/journal.pone.0104263)
Supplement: Appendix S1 — Detail of anti-diabetes used in three hospitals in this study. (DOCX) [file pone.0104263.s001.docx]

**Appendix S1. Detail of anti-diabetes used in three hospitals in this study**

Anti-diabetes medication used regularly by diabetic patients in three hospitals (Taipei Medical University Hospital, Wan-Fang Hospital and Shuang-Ho Hospital) during the period from 2007 to 2011.

| **Drug name** | **ATC code** |
| --- | --- |
| Metformin | A10BA02 |
| Glyburide | A10BB01 |
| Glipizide | A10BB07 |
| Gliclazide | A10BB09 |
| Glimepiride | A10BB12 |
| Acarbose | A10BF01 |
| Rosiglitazone | A10BG02 |
| Pioglitazone | A10BG03 |
| Sitagliptin | A10BH01 |
| Vildagliptin | A10BH02 |
| Repaglinide | A10BX02 |
| Nateglinide | A10BX03 |

**Insulin**

| **Drug name** | **ATC code** |
| --- | --- |
| Insulin Aspart | A10AB05 |
| Insulin Aspart | A10AD30 |
| Insulin Glargine | A10AE04 |
