# Supplementary material for: Using Hemoglobin A1C as a Predicting Model for Time Interval from Pre-Diabetes Progressing to Diabetes
Source: PLoS One. 2014 Aug 5;9(8):e104263. doi: 10.1371/journal.pone.0104263 (PMC4122428; doi:10.1371/journal.pone.0104263)
Supplement: Appendix S2 — Overall study design. (DOCX) [file pone.0104263.s002.docx]

**Appendix S2. Overall study design**

January 2007 – June 2011 three hospitals claimed data had a total 49,648 patients with HbA1c laboratory examinations

- Excluded 39,762 patients who had used anti-diabetes medications before HbA1c examinations in hospitals
- Excluded 8,607 patients who had once time HbA1c examinations in hospitals

1,279 patients were selected for applying a linear regression trend-line that calculated the trend of HbA1c tests and estimated the risk diabetes intervals

27 patients were excluded which found at extreme interval of 2.5 % at both sides

1,252 patients were categorized in three groups (low risk, increased risk and diabetes) based on the baseline of HbA1c examination
